# MicroRNA miR-502-5p inhibits ovarian cancer genesis by downregulation of GINS complex subunit 2

**DOI:** 10.1080/21655979.2021.1946347

**Published:** 2021-07-21

**Authors:** Lili Zhan, Jing Yang, Yang Liu, Yanxiang Cheng, Hua Liu

**Affiliations:** a Dept of Obstetrics and Gynecology, Renmin Hospital of Wuhan University, Wuhan, Hubei Province, P.R.C; b Dept of Orthopedics, Renmin Hospital of Wuhan University, Wuhan, Hubei Province, P.R.C; c Dept of Reproductive Medicine Center, Renmin Hospital of Wuhan University, Wuhan, Hubei Province, P.R.C

**Keywords:** miR-502-5p, GINS2, ovarian cancer, proliferation, apoptosis, migration

## Abstract

Ovarian cancer (OC) is one of the most common malignancies with high incidence and mortality and the eighth most common cancer-associated mortality in women worldwide. Aberrant expression of the GINS complex subunit 2 (GINS2) gene and miR-502-5p has been associated with cancer progression. This study aims to investigate the specific molecular mechanism of the miR-502-5p-GINS2 axis in OC. GINS2 and miR-502-5p expression in OC tissues and cell lines was measured using RT-qPCR. Next, we investigated the interaction between miR-502-5p and GINS2 using a luciferase assay. The role of the miR-502-5p-GINS2 axis was detected by assessing cell proliferation, migration, and apoptosis levels, such as caspase-3 activity and caspase-3 protein expression, in the OC cell lines CaOV3 and SKOV3, respectively. MiR-502-5p expression was decreased, and GINS2 expression was dramatically elevated in OC tissues and cells. Upregulation of miR-502-5p expression repressed cellular proliferation and migration levels but increased the cellular apoptosis level. GINS2 overexpression enhanced the proliferation and migration levels but hampered OC cell apoptosis. Moreover, miR-502-5p inhibited GINS2 expression and suppressed OC tumorigenesis. miR-502-5p targeting GINS2 suppressed OC progression by inhibiting cell growth and promoting cell apoptosis. Hence, we provide a comprehensive understanding of OC involving both miR-502-5p and GINS2, which might be effective therapeutic targets for OC patients.

## Graphical Abstract

**Figure:**
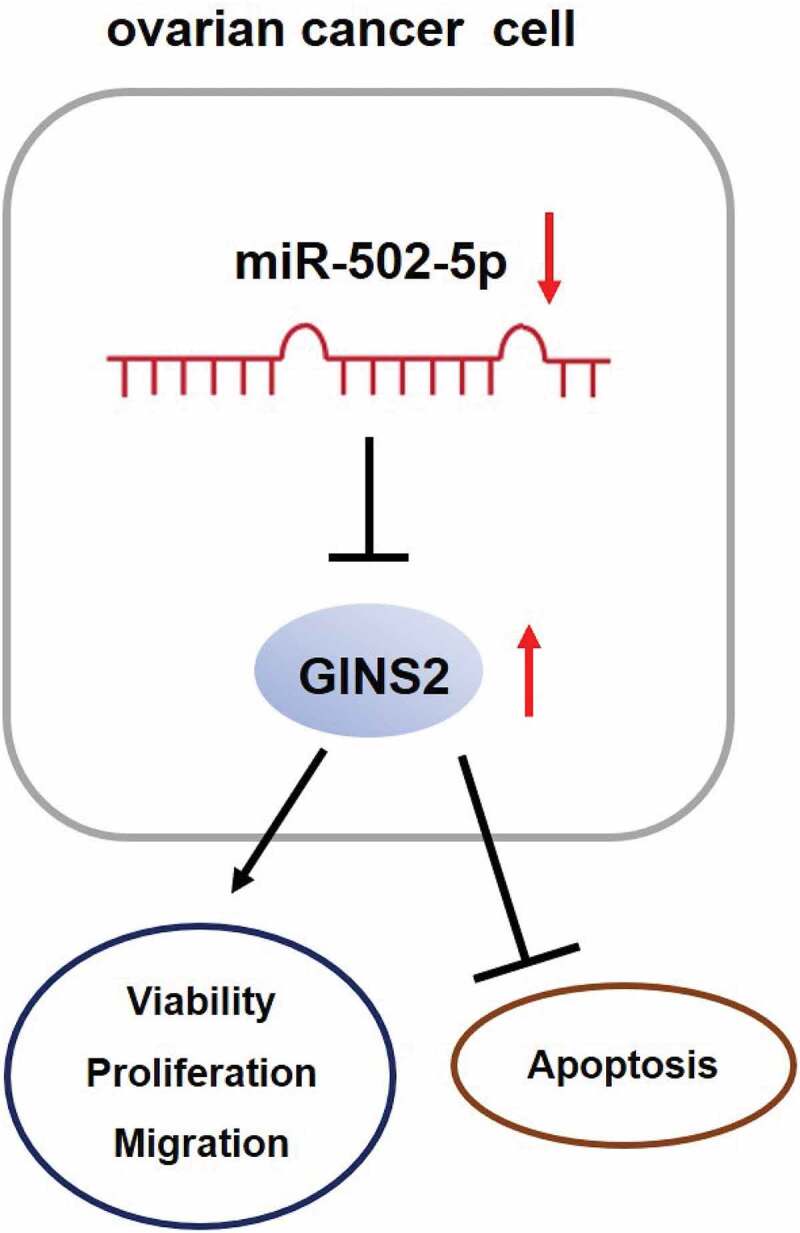

## Introduction

Ovarian cancer (OC) is one of the most common malignancies worldwide, with 1.6% incidence and 1.9% mortality, and ranks as the eighth most common cancer-associated mortality in women [1]. Owing to the nonspecific symptoms of OC, the diagnosis and therapeutic strategies remain unsatisfactory [2]. Studies have shown that inherited mutations in genes are linked to the development of OC [3]. Therefore, it is essential to comprehensively investigate the genetic changes and pathogenesis of OC.

MicroRNAs (miRNAs) are small RNAs of 20–24 nucleotides that silence mRNAs either through mRNA degradation or by preventing mRNA translation [4,5]. Evidence has shown that certain miRNAs contribute to cancer cellular biological processes, thus serving as tumor suppressors or oncogenes [6,7]. Moreover, miRNAs have long been shown to regulate the occurrence and development of OC. For instance, miR-552 was upregulated in OC, resulting in the promotion of growth and metastasis of OC cells [8]. MiR-34a-5p reduced the proliferation and tumorigenicity of cisplatin-resistant OC cells in nude mice, blocked the G1 cell cycle, and induced apoptosis [9]. In addition, miR-502-5p has been reported to be associated with different types of cancers, such as gastric, breast, and bladder cancers [10–12]. Upregulation of miR-502-5p expression could prevent cell growth and metastasis by targeting different genes and activating various signaling pathways in these cancers. However, only two studies have suggested that miR-502 expression is decreased in OC patients [13]. miR-502 overexpression can repress interferon alpha-inducible protein 27 (IFI27)-induced tumorigenicity, migration, and drug resistance [13]. Furthermore, the polymorphism of the miR-502 binding site is associated with the carcinogenic mechanism of epithelial OC [14]. Nonetheless, the molecular mechanisms underlying the role of miR-502-5p in OC development remain unknown.

GINS complex subunit 2 (GINS2) serves as an oncogene in cancer genesis by enhancing cell growth and repressing cell apoptosis in various cancers, including thyroid, lung, and pancreatic cancers [15–17]. It has also been associated with the progression of OC by regulating cell growth and apoptosis [18,19]. However, the biological function of the interaction between miR-502-5p and GINS2 in OC requires further study.

To elucidate the mechanism of the occurrence and development of OC, we used GSE119055 data and previous literature to screen out the miRNAs of interest in OC, namely miR-502-5p. GEPIA OC data and TargetScan algorithm screened GINS2 as a target gene candidate for miR-502-5p. Therefore, we sought to elucidate the effects of miR-502-5p and GINS2 on OC. miR-502-5p expression decreased and GINS2 expression increased in OC. Further functional experiments confirmed that the tumor suppressive effect of miR-502-5p on OC was realized through targeted GINS2 inhibition. Our study is the first to reveal the effect and mechanism of miR-502-5p/GINS2 on OC, which may provide a new direction for the treatment of OC involving both genes.

## Materials and methods

### Tissue samples, cell lines, and cell transfection

Human OC tissue specimens (n = 30) and normal tissues (n = 30) were collected from OC patients in our hospital after obtaining written informed consent and approval from our hospital ethics committee. The patients’ baseline data are presented in Table 1. Human OC cell lines (SKOV3, CaOV3, and OVCAR3) and normal ovarian epithelial cells (HOSEpiC) were obtained from ATCC (VA, USA) and maintained in RPMI-1640 medium (Gibco, MA, USA) with 10% fetal bovine serum (FBS; Gibco) at 37°C with 5% CO_2_. For transfection, miR-502-5p mimic (100 nM) and the inhibitor (75 nM), GINS2-overexpression plasmid (GINS2-OE; 2 μg/mL), and the corresponding negative controls (NC) were obtained from GenePharm (Shanghai, China), transfected into CaOV3 and SKOV3 cells using Lipofectamine 3000 (Invitrogen, CA, USA), and cultured for 48 h at 37°C for other experiments.

**Table 1.** The baseline characteristics of 30 ovarian cancer patients

Categories	Patients (Total n = 30)
Age (years)	
≤60	17(56.67%)
>60	13(43.33%)
Primary tumor expansion	
TX	1(3.33%)
T1	7(23.33%)
T2	4(13.33%)
T3	18(60.00%)
Histology	
Serous	17(56.67%)
Clear cell	3(10.00%)
Endometrioid	6(20.00%)
Mucinous	4(13.33%)
Nodal status	
pNX	12(40.00%)
pN0	9(30.00%)
Pn1	9(30.00%)
Distant metastasis	
pMX	28(93.33%)
pM0	1(3.33%)
pM1	1(3.33%)
Grading serous	
Low	7(23.33%)
High	23(76.67%)
Grading endometrioid	
G1	1(16.67%)
G2	2(33.33%)
G3	3(50.00%)
Grading mucinous	
G1	1(25.00%)
G2	3(75.00%)
G3	0(0%)
Grading clear cell	
G3	3(100.00%)
FIGO	
I	5(16.67%)
II	3(10.00%)
III	21(70.00%)
IV	1(3.33%)

### RNA isolation and RT-qPCR analysis

Total RNA from OC tissues and cells was extracted using TRIzol reagent (Invitrogen, CA, USA) and transcribed to cDNA using the PrimeScript First Strand cDNA Synthesis Kit (Cat#: #6110A, Takara, Kyoto. Japan). RT-qPCR was performed using SYBR Premix Ex Taq (Cat#: #RR420A Takara). Total miRNA from the OC tissues and cell lines was isolated using the miRNeasy Mini Kit (Cat#: #217,004, QIAGEN, Hilden, Germany) and transcribed to cDNA using miScript II RT Kit (Cat#: #218,161, QIAGEN). RT-qPCR was performed using the miScript SYBR Green PCR Kit (Cat#: #218,075, QIAGEN). The 2-^ΔΔCt^ method was applied to obtain the results of GINS2 expression normalized to GAPDH and miR-502-5p levels normalized to U6. All primer sequences are listed in Table 2.

**Table 2.** Primer sequences

Genes	Primer sequences
miR-502-5p	Forward:5ʹ-CGGGCATCCTTGCTATCTG-3ʹ Reverse:5ʹ-CAGCCACAAAAGAGCACAAT-3’
GINS2	Forward:5ʹ-CAGAAATGTCGCCTGCTCC-3ʹ Reverse:5ʹ-GGATTTCGTCTGCCTTCG-3’
GAPDH	Forward:5ʹ-TGACTTCAACAGCGACACCCA-3ʹ Reverse:5ʹ-CACCCTGTTGCTGTAGCCAAA- 3’
U6	Forward:5ʹ-CTCGCTTCGGCAGCACA- 3ʹ Reverse:5ʹ-AACGCTTCACGAATTTGCGT- 3’

### CCK-8 assay

This assay was performed as previously described [20]. CaOV3 and SKOV3 cells (5 × 10^3^ cells) were seeded into 96-well plates and cultured for 0, 24, 48, and 72 h, and cell viability was measured at the four time points using the CCK-8 kit (Cat#: K1018; APExBIO, TX, USA). Briefly, 10 µL CCK-8 solution was added to the plate and incubated for 2 h at 37°C. Finally, OD450 was measured on a multimode-plate reader (Thermo Fisher, MA, USA).

### BrdU assay

The BrdU assay was performed according to the BrdU Cell Proliferation Assay Kit (Cat#: 6813, CST, MA, USA), as previously described [21]. CaOV3 and SKOV3 cells (5 × 10^3^ cells) were seeded into 96-well plates and cultured overnight. After washing twice, the cells were denatured and labeled with 10 µL of 10X BrdU solution antibody for 2 h. Then, the medium was removed, and the HRP-conjugated secondary antibody solution was added into each well and incubated for 1 h. Finally, OD450 was performed on a multimode-plate reader (Thermo Fisher).

### Apoptosis assay

The CaOV3 and SKOV3 cell apoptosis assays were performed using a caspase-3 activity kit (Cat#: 5723, CST), as previously described [22]. The cells were collected, washed twice with PBS, and treated with cell lysis buffer for 10 min. Subsequently, the cell lysate was treated with a loading solution (100 µL/well) at 37°C for 2 h. Finally, OD405 was performed on a multimode-plate reader (Thermo Fisher).

### Transwell assay

A 24-well plate chamber (Cat#: #3244, Corning, NY, USA) was used for cell migration measurements according to a previous study [23]. The lower chamber was supplemented with 10% FBS medium, and CaOV3 and SKOV3 cell suspensions (2 × 10^5^ cells) without FBS were added to the upper chamber. After 24 h of culture, the cells that passed through the filter were fixed with 4% paraformaldehyde and stained with 0.1% crystal violet (C0775, Sigma-Aldrich, MO, USA). We then randomly captured five different fields from each well using a light microscope. Three randomly selected fields were observed under a microscope.

### Luciferase assay

The pmiRGLO GINS2 3′-UTRs WT vector and pmiRGLO GINS2 3′-UTR MUT vector were provided by GenePharma (Shanghai, China). CaOV3 and SKOV3 cells were treated with pmiRGLO GINS2 3′-UTR WT or MUT vectors and either miR-NC or miR-502-5p using Lipofectamine 3000 for 72 h. Then, the firefly and Renilla luciferase activities were measured using the Luciferase Assay Kit (Cat#: #16,185, Thermo Fisher). Relative luciferase activities were normalized to firefly luciferase, according to a previous study [24].

### RNA-pull down analysis

This assay was performed as previously described [20]. Biotin-labeled miR-502-5p (Bio-miR-502-5p) and miR-NC (Bio-NC) were obtained from Thermo Fisher. The CaOV3 and SKOV3 cell lysate suspensions were incubated with streptavidin beads (Cat#: #88,817, Thermo Fisher) conjugated with Bio-miR-502-5p or Bio-NC at 4°C overnight. Subsequently, the eluted solution was purified using the RNeasy Mini Kit (Cat# 74,104, QIAGEN). Finally, GINS2 enrichment was performed using RT-qPCR.

### Western blotting analysis

Western blotting was performed as previously described [25]. Equal quantities of protein from CaOV3 and SKOV3 cells were isolated using RIPA buffer (Cat#: #20-188, Sigma-Aldrich), and the total protein concentration was determined using a BCA protein assay kit (Beyotime, Shanghai, China). The samples were loaded (20 μg/lane) on 10% SDS polyacrylamide gels and transferred to PVDF membranes. After blocking and washing the membrane, anti-GINS2 (1:1000, Cat#: ab197123, Abcam, Cambridge, UK), anti-cleaved caspase-3 (1:500, Cat#: ab2302, Abcam), anti-total caspase-3 (1:10,000, Cat#: ab32499, Abcam), and anti-GAPDH (1:2000, Cat#: ab9485, Abcam) were used to incubate the membranes overnight at 4°C. Then, the second antibody Anti-HRP-Rabbit was used for 1 h incubation at 25°C. ECL reagents (Bio-Rad, CA, USA) were used to develop the protein bands. Relative GINS2 protein expression was normalized to that of GAPDH.

### Statistical analysis

All data were analyzed using paired Student’s *t*-tests and one-way analysis of variance with Dunnett’s post hoc test for two groups and multiple groups analysis, respectively, using GraphPad 8.0 software (GraphPad, CA, USA). The results are shown as the mean ± SD from three independent experiments. In addition, Pearson correlation analysis was used to study the relationship between GINS2 and miR-502-5p in OC tissues. Statistical significance was set at *P* < 0.05.

## Results

### miR-502-5p might be a significant participant in OC by suppressing GINS2

**Figure 1.**
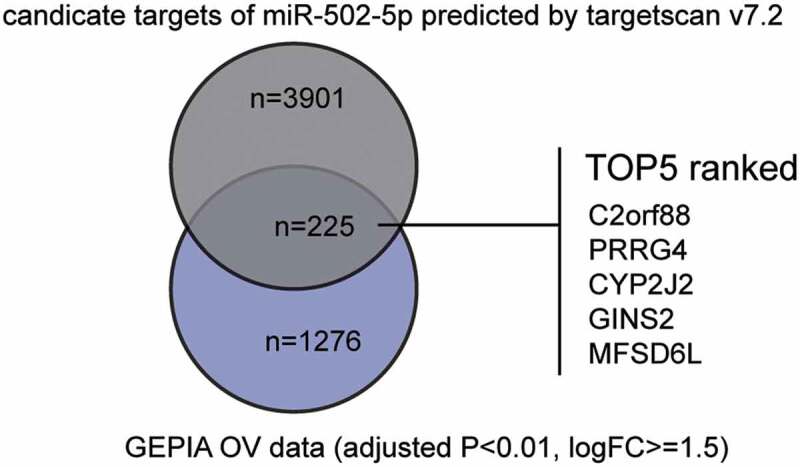
miR-502-5p might be a significant participant in OC by suppressing GINS2

To identify the influencing factors in OC, we first screened out the interesting miRNA (miR-502-5p) and mRNA (GINS2) using bioinformatics analysis. By analyzing GSE119055 data using the GEO2R algorithm (https://www.ncbi.nlm.nih.gov/geo/geo2r/), we identified significantly downregulated miRNAs in OC. By listing them in ascending order, we found that miR-502-5p, the top third most downregulated miRNA, has not been studied in OC but has been studied in other cancers, such as gastric [10,26,27], breast [11], colon [28], and bladder [12] cancers as tumor suppressors. Differentially expressed genes from GEPIA OC data were obtained and intersected with the candidate genes that could be targeted by miR-502-5p, which were predicted by the TargetScan algorithm. In total, 225 genes were identified and ranked according to their context++ score (Figure 1). Among the five with the lowest context++ scores, GINS2 was once reported to be a cancer driver in OC [19] and was associated with worse prognosis in OC patients [18]. More thorough studies on GINS2 are required.

The Venn diagram showing the common results of the prediction results of miR-502-5p and the DEGs from GEPIA OC data (http://gepia2.cancer-pku.cn/#degenes).The top 5 most ranked genes according to their context++ score in targetscan algorithm were listed.

### MiR-502-5p suppressed cell progression in OC

**Figure 2.**
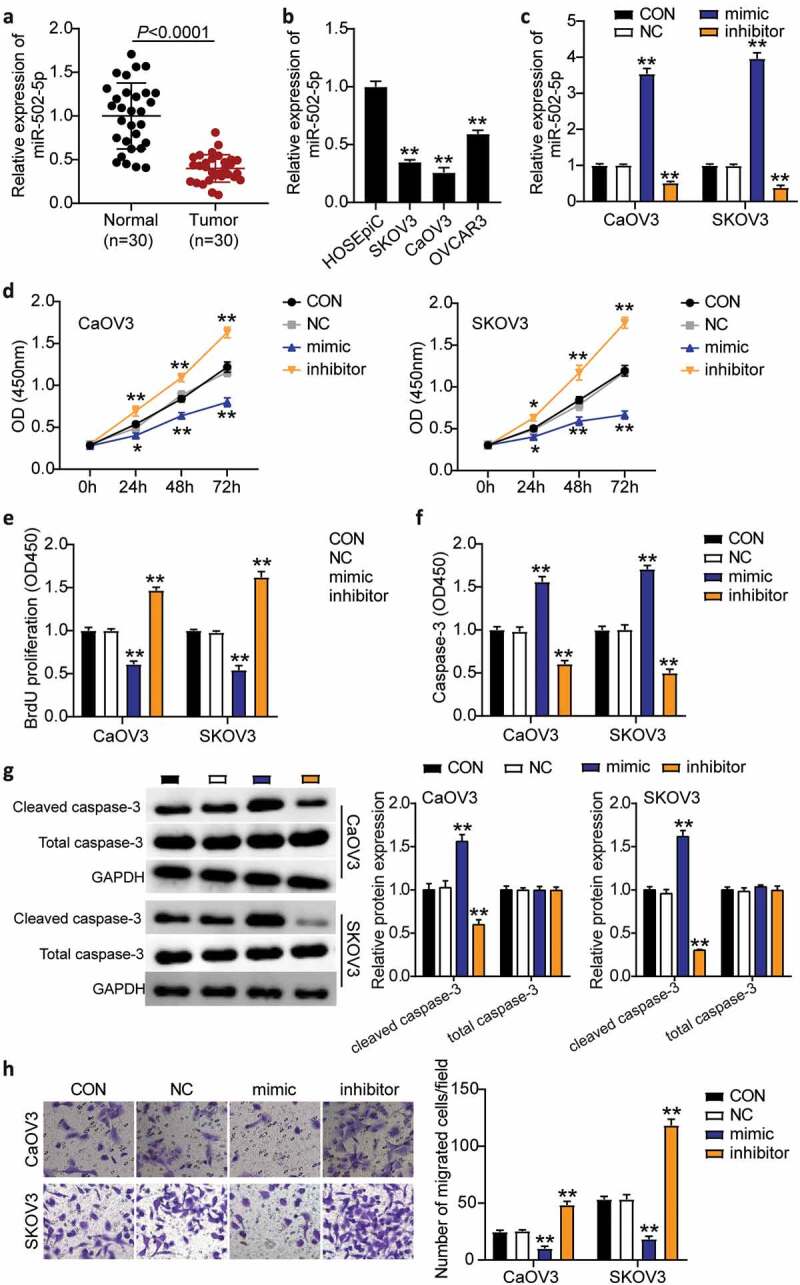
MiR-502-5p inhibited cell proliferation and migration, and promoted cell apoptosis in OC

To evaluate the role of miR-502-5p in OC, we detected miR-502-5p expression in OC tissues and cell lines, explored the effects of miR-502-5p on the proliferation, migration, and apoptosis of OC cells in vitro using biological function experiments, and clarified the antitumor effect of miR-502-5p in OC. miR-502-5p expression was downregulated by more than 50% in tumor tissues compared to normal tissues (Figure 2a). OC cell lines (SKOV3, CaOV3, and OVCAR3) showed dramatically decreased miR-502-5p expression compared to normal ovarian epithelial cells (HOSEpiC). We chose the CaOV3 and SKOV3 cell lines for subsequent experiments because they showed the lowest miR-502-5p expression (Figure 2b). Next, we transfected the miR-502-5p inhibitor, mimic, and NC into CaOV3 and SKOV3 cell lines. The results showed that the mimic groups upregulated miR-502-5p expression by approximately 4-fold, while the inhibitor groups showed 50% miR-502-5p expression compared to control cells in both cell types (Figure 2c). The mimic groups showed significantly increased cell viability, but the inhibitor groups showed decreased cell viability compared to control cells in both cell lines (Figure 2d). In addition, the mimic groups reduced cell proliferation by 40%, while the inhibitor groups enhanced cell proliferation by approximately 1.5-fold compared to control cells in both CaOV3 and SKOV3 cells (Figure 2e). Furthermore, the mimic groups enhanced cell apoptosis by approximately 1.5-fold, while the inhibitor groups suppressed cell apoptosis by approximately 50% compared with control cells in both CaOV3 and SKOV3 cells (figure 2f). Similarly, cleaved caspase-3 protein levels increased in the mimic groups and decreased in the inhibitor groups compared to the control groups (Figure 2g). Moreover, the mimic groups inhibited cell migration levels by 60%, while the inhibitor groups enhanced cell migration by approximately 2-fold compared to control cells in both cell types (Figure 2h). Collectively, these results indicated that miR-502-5p inhibited cell growth and elevated cell apoptosis in OC.

(a) RT-qPCR detection of miR-502-5p expression in OC tissues and normal tissues. (b) Measurement of miR-502-5p expression in OC cells lines (SKOV3, CaOV3, and OVCAR3) and normal ovarian epithelial cell (HOSEpiC). (c) Measurement of miR-502-5p expression in CaOV3 and SKOV3 cells transfected with NC, miR-502-5p inhibitor and miR-502-5p mimic by RT-qPCR. (d) Cell viability was detected in CaOV3 and SKOV3 cells transfected with miR-502-5p mimic, miR-502-5p inhibitor and NC by CCK-8 assay. (e) Cell proliferation was detected in CaOV3 and SKOV3 cells transfected with miR-502-5p mimic, miR-502-5p inhibitor and NC by BrdU assay. (f) Cell apoptosis level was determined in CaOV3 and SKOV3 cells transfected with NC, miR-502-5p inhibitor and miR-502-5p mimic. (g) The expression of cleaved and total caspase-3 protein expression was determined in CaOV3 and SKOV3 cells transfected with NC, miR-502-5p inhibitor and miR-502-5p mimic. (h) Cell migration level was determined in CaOV3 and SKOV3 cells transfected with NC, miR-502-5p inhibitor and miR-502-5p mimic. *, *P* < 0.05; **, *P* < 0.001. NC, negative control; mimic, miR-502-5p mimic; inhibitor, miR-502-5p inhibitor.

### miR-502-5p targeted GINS2 and inhibited the expression of GINS2

**Figure 3.**
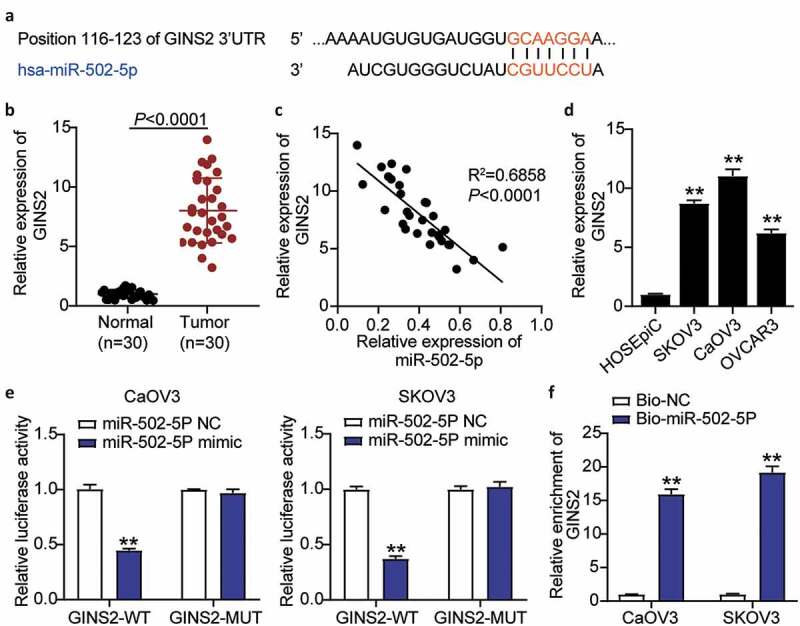
MiR-502-5p targeting GINS2 and inhibited the expression of GINS2

To understand the mechanism of action of miR-502-5p in OC, luciferase and RNA-pull down assays were performed to analyze whether miR-502-5p targeted GINS2 and inhibited GINS2 expression. TargetScan Human 7.2 analysis revealed the binding sequence of GINS2 and miR-502-5p (Figure 3a). GINS2 expression in OC tumor tissues was upregulated by approximately 8-fold compared to that in normal tissues (Figure 3b). Meanwhile, a negative correlation between miR-502-5p expression and GINS2 expression was observed in OC tumor tissues (Figure 3c). In addition, GINS2 expression was dramatically increased in OC cell lines (SKOV3, CaOV3, and OVCAR3) compared to normal ovarian epithelial cells (HOSEpiC) (Figure 3d). Next, we transfected pmiRGLO GINS2 3′-untranslated regions (3′-UTRs) WT vectors or pmiRGLO GINS2 3′-UTR MUT vectors and miR-502-5p-NC or miR-502-5p-mimic into CaOV3 and SKOV3 cell lines. The luciferase activities of both cell lines with wild-type GINS2 3′UTR and miR-502-5p-mimic dramatically decreased by 50%, but no difference was observed in the mutant GINS2 3′UTR groups (Figure 3e). The RNA-pull down assay showed a strong binding effect between GINS2 and miR-502-5p in both CaOV3 and SKOV3 cells (figure 3f). Taken together, these results revealed that miR-502-5p significantly suppressed GINS2 expression.

(a) Bioinformatics analysis showed the binding sequence of miR-502-5p and GINS2 3ʹ-UTR. (b) RT-qPCR detection of expression of GINS2 in the OC tissues and normal tissues. (c) Correlation analysis between the miR-502-5p expression and GINS2 expression in the OC tumor tissues. (d) Measurement of GINS2 expression in OC cells lines (SKOV3, CaOV3, and OVCAR3) and normal ovarian epithelial cell (HOSEpiC). (e) Dual luciferase assay was performed in cells co-transfected with plasmids GINS2-WT or GINS2-MUT and miR-NC or miR-502-5p mimic in CaOV3 and SKOV3 cells. (f) RT-qPCR detection of expression of GINS2 in CaOV3 and SKOV3 cells transfected with Bio- miR-502-5p NC or Bio- miR-502-5p mimic. *, *P* < 0.05; **, *P* < 0.001. WT, wild-type; MUT, mutant; NC, negative control.

### miR-502-5p targeting GINS2 hampered the progression of OC

**Figure 4.**
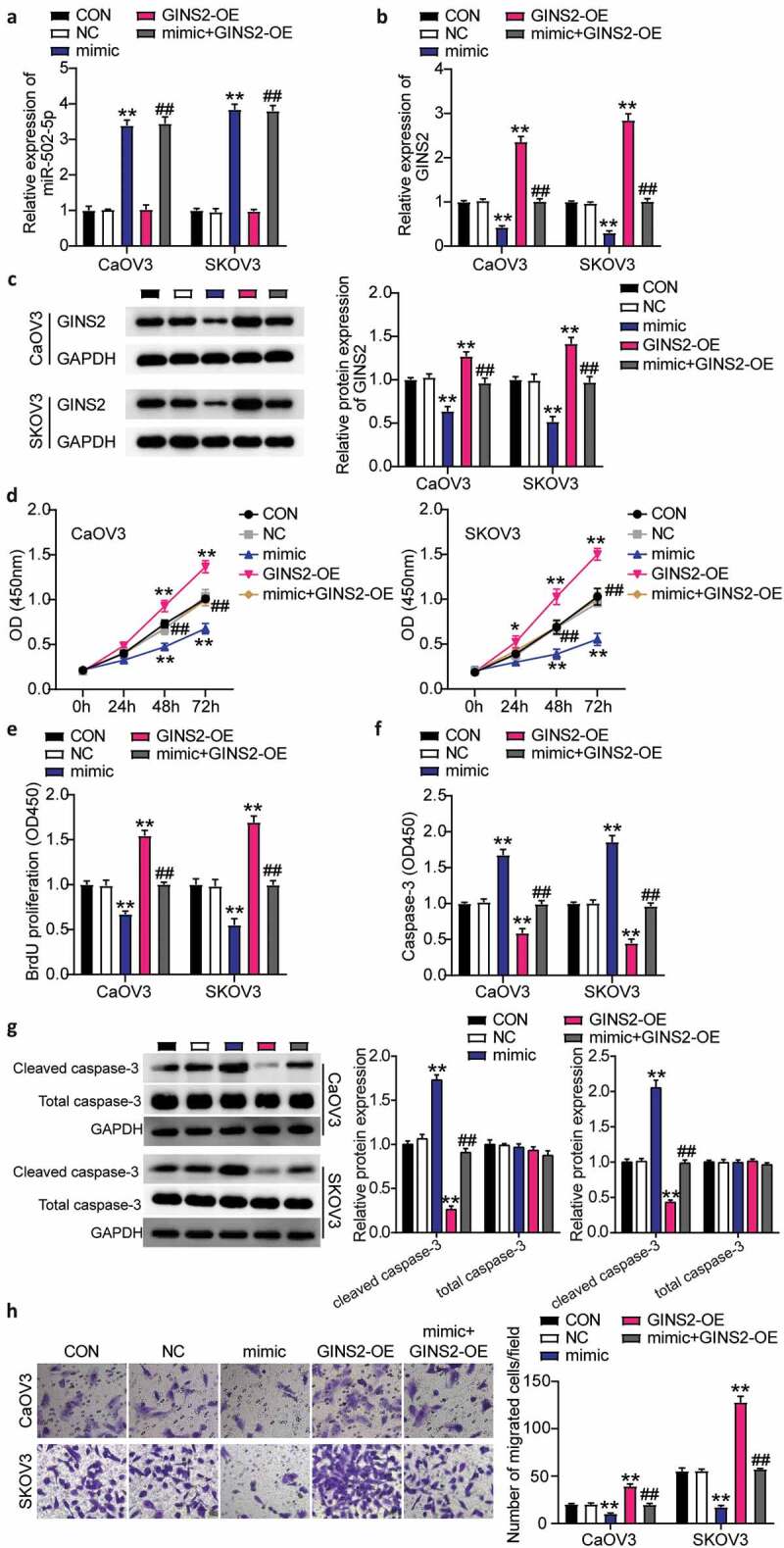
MiR-502-5p targeting GINS2 repressed the progression of OC

To further investigate the effect of miR-502-5p/GINS2 on the biological function of OC cells, CCK-8, BrdU, caspase-3 activity, and transwell assays were used to detect the changes in proliferation, apoptosis, and migration. It was shown that miR-502-5p targeting GINS2 inhibited the malignant proliferation of OC cells. First, we transfected the GINS2 overexpression plasmid and mimicked CaOV3 and SKOV3 cells. miR-502-5p expression was enhanced by approximately 4-fold in the mimic groups and mimic+ OE groups, while the expression level in the OE-GINS2 groups was the same as that in control cells in both cell lines (Figure 4a). Meanwhile, GINS2 expression in the mimic groups decreased by 80% and that in the OE- GINS2 groups increased approximately 2-fold compared to control cells. The mimic+ OE groups showed the same expression level as the control cells in both CaOV3 and SKOV3 cells (Figure 4b). Western blot analysis revealed approximately 1.3-fold increase in GINS2 protein expression in the OE-GINS2 groups and approximately 60% decrease in GINS2 protein expression in the mimic groups compared to control cells in both CaOV3 and SKOV3 cells. However, the expression in the OE-GINS2 and miR-502-5p-mimic co-transfection group is comparable to that in the control groups (Figure 4c). Next, we found that the OE-GINS2 groups showed significantly higher cell viability than the control cells, while the effect of OE-GINS2 on cell viability diminished in the mimic+ OE groups (Figure 4d). The OE-GINS2 groups showed approximately 1.5-fold increase in cell proliferation compared to control cells, while the effect diminished in the mimic+ OE groups (Figure 4e). Furthermore, the OE-GINS2 groups show a 50% reduction in cell apoptosis compared to control cells, while the effect diminished in the mimic+ OE groups (figure 4f). Moreover, GINS2 overexpression reduced cleaved caspase-3 protein expression and reversed the effect of increased cleaved caspase-3 expression in miR-502-5p-mimic groups (Figure 4g). Finally, the OE-GINS2 groups show an approximately 2-fold increase in cell migration compared to control cells, while the effect diminished in the mimic+ OE groups (Figure 4h). Hence, the results clarified that miR-502-5p targeting GINS2 hampered the progression of OC.

(a) Measurement of miR-502-5p expression in CaOV3 and SKOV3 cells transfected with NC, OE- GINS2, mimic, and OE+ mimic by RT-qPCR. (b) Measurement of GINS2 expression in CaOV3 and SKOV3 cells transfected with NC, OE- GINS2, mimic, and OE+ mimic by RT-qPCR. (c) Measurement of GINS2 protein expression in CaOV3 and SKOV3 cells transfected with NC, OE- GINS2, mimic, and OE+ mimic by western blot. (d) Cell viability was detected in CaOV3 and SKOV3 cells transfected with NC, OE- GINS2, mimic, and OE+ mimic by CCK-8 assay. (e) Cell proliferation was detected in CaOV3 and SKOV3 cells transfected with NC, OE- GINS2, mimic, and OE+ mimic by BrdU assay. (f) Cell apoptosis was determined in CaOV3 and SKOV3 cells transfected with NC, OE- GINS2, mimic, and OE+ mimic by Caspase-3 apoptosis detection kit. (g) The expression of cleaved and total caspase-3 protein expression was determined in CaOV3 and SKOV3 cells transfected with NC, OE- GINS2, mimic, and OE+ mimic. (h) Cell migration level was determined in CaOV3 and SKOV3 cells transfected with NC, OE- GINS2, mimic, and OE+ mimic. *, *P* < 0.05; **, *P* < 0.001 compared with CON; ^#^, *P* < 0.05; ^##^, *P* < 0.001 mimic+GINS2-OE compared with GINS2-OE. NC, negative control; OE- GINS2, overexpression-GINS2; mimic, miR-502-5p mimic. OE+ mimic, overexpression-GINS2+ miR-502-5p mimic.

## Discussion

In this study, we showed that miR-502-5p expression was clearly reduced, while GINS2 expression was dramatically elevated in OC tissues and cells. In addition, miR-502-5p inhibiting GINS2 functionally suppressed cell growth and enhanced cell apoptosis in OC.

Accumulating evidence suggests that miR-502-5p attenuates cell growth and promotes cell apoptosis in numerous cancers, particularly gastric cancer [10,26,29,30]. One study demonstrated that miR-502-5p repressed gastric cancer development by suppressing PD-L1 expression [10]. In addition, miR-502-5p inhibits cell growth by inhibiting SP1 in gastric cancer cells [26]. miR-502-5p inhibited the expression of CCND1, NOP14, and DNMT3B, the targets of miR-502-5p, by reducing cell migration in bladder cancer [31]. Moreover, miR-502-5p reduces cell growth by downregulating the expression of TRAF2 in breast cancer cells [11]. Notably, one study indicated that miR-502 inhibited EMT and ovarian tumorigenicity and stemness by inhibiting the expression of interferon alpha-inducible protein 27 [13]. In this study, we demonstrated that miR-502-5p expression was significantly reduced in OC tissues and cells. Upregulation of miR-502-5p repressed cell growth, while boosting apoptosis in the OC cells, CaOV3 and SKOV3. Furthermore, miR-502-5p directly inhibited GINS2 expression, which reduced the tumorigenic effect of GINS2 on OC cells.

In recent decades, GINS2 has been involved in the development of various cancers, such as pancreatic, lung, and thyroid cancers [15,17,32]. Zhang *et al*. demonstrated that GINS2 elevated cell growth and cell cycle progression by activating the MAPK/ERK pathway in pancreatic cancer cells [17]. In addition, downregulation of GINS2 repressed cell growth and enhanced cell apoptosis by inhibiting the p53/GADD45A pathway in non-small cell lung cancer [32]. GINS2 promotes cell proliferation and inhibits cell apoptosis by regulating CITED2 and LOXL2 in thyroid cancer [15]. As mentioned above, GINS2 promotes cell proliferation but suppresses apoptosis of OC cells [19,33]. Yan *et al*. found that GINS2 is upregulated in OC cells. Furthermore, knockdown of GINS2 dramatically repressed SKOV-3 cell growth and boosted cell cycle arrest and cell apoptosis [19]. In this study, we demonstrated that GINS2 expression was increased in ovarian tissues and cells. GINS2 overexpression significantly facilitated cell growth but suppressed apoptosis in CaOV3 and SKOV3 cells, and this effect was diminished by miR-502-5p.

Previous studies have shown that miRNAs hybridized with complementary sequences in mRNA and silenced genes by destabilizing mRNA or preventing translation of mRNA [34]. Accumulated evidence has also revealed that some miRNAs regulate OC progression by targeting mRNA. For instance, Bi et al. [35] found that miR-125b negatively regulated S100A4 to promote cell apoptosis and reduce cell survival, migration, tumor growth, and lung metastasis. Cai et al. [36] studied the inhibition of proliferation, migration, and invasion of OC cells by miR-330-3p targeting RIPK4. In this study, GINS2 was found to be a potential target of miR-502-5p, and the upregulation of miR-502-5p inhibited the proliferation and migration of ovarian cancer cells through GINS2. This is similar to previous studies on the regulatory mechanism of miRNA-mRNA in OC [35,36]. Therefore, this study proposed a novel mechanism by which miR-502-5p/GINS2 affects OC.

However, there are certain limitations to this study. First, the specific signaling pathways and mouse model confirmation of the miR-502-5p-GINS2 axis in OC need further exploration. Second, although the differential expression of miR-502-5p has been proven using bioinformatics analysis and qRT-PCR in patient tissues, the direct association between OC and miR-502-5p could not be confirmed. In the future, it will be necessary to analyze the correlation between miR-502-5p and OC in terms of clinicopathological changes and patient prognosis.

## Conclusion

Overall, this study revealed that miR-502-5p significantly decreased and GINS2 significantly increased in OC, and miR-502-5p hampered OC progression by repressing GINS2 expression, including suppression of viability, proliferation, migration, and elevated apoptosis of cells. Therefore, our study provides a comprehensive understanding of the miR-502-5p-GINS2 axis in OC, which may offer a new direction for the treatment of OC involving both miR-502-5p and GINS2.

## Data Availability

The datasets used and/or analyzed during the current study are available from the corresponding author on reasonable request.

## Highlights

miR-502-5p was downregulated and GINS2 was upregulated in OC tissues and cells.

miR-502-5p upregulation inhibited the proliferation and migration of OC cells and induced apoptosis.

GINS2 overexpression increased the proliferation and migration of OC cells and inhibited apoptosis.

GINS2 was confirmed to be the target of miR-502-5p.
